# National Association of Dental Faculties
*We are indebted to Dr. James W. White, editor of *Dental Cosmos*, for advanced press sheets of these proceedings.


**Published:** 1886-08

**Authors:** 


ARTICLE IV.

### NATIONAL ASSOCIATION OF DENTAL FACULTIES.*

The National Association of Dental Faculties held its third annual meeting in the Park Theatre, Niagara Falls, commencing Monday, August 2, 1886, President C. N. Peirce, Philadelphia, in the chair.

The following colleges were represented:

*Pennsylvania College of Dental Surgery.*—C. N. Peirce.

*Chicago College of Dental Surgery.*—T. W. Brophy, A. W. Harlan, F. H. Gardiner, J. A. Swasey, and L. P. Haskell.

*Missouri Dental College.*—W. H. Eames and A. H. Fuller.

*Boston Dental College.*—J. A. Follett.

*Philadelphia Dental College.*—S. H. Guilford.

*University of Pennsylvania, Dental Department.*—James Truman.

*Baltimore College of Dental Surgery.*—R. B. Winder.

*Dental Department, State University of Iowa.*—L. C. Ingersoll and A. O. Hunt.

*Dental College of the University of Michigan.*—J. Taft and J. A. Watling.

*Ohio College of Dental Surgery.*—H. A. Smith.

*New York College of Dentistry.*—Frank Abbott.

*Kansas City Dental College.*—C. B. Hewitt.

The following additional colleges were admitted to membership:

*Minnesota Hospital College, Dental Department.*—W. A. Spalding.

*Vanderbilt University, Dental Department.*—W. H. Morgan.

---

*We are indebted to Dr. James W. White, editor of *Dental Cosmos*, for advanced press sheets of these proceedings.

*University of California, Dental Department.*—S. W. Dennis.

*Harvard University, Dental Department.*—Thos. Fillebrown.

*Dental Department of St. Paul Medical College.*—L. W. Lyon.

Dr. Winder, chairman of the committee on text-books, reported verbally that so much opposition to the plan submitted had been expressed last year that he had concluded to let the matter rest until this meeting so as to get the views of all the schools possible. Since he arrived here he had learned that a much larger number of the profession were in favor of the idea than appeared at the meeting in Chicago. A work is being prepared under the editorial supervision of Dr. Wilbur F. Litch, but it is an encyclopœdia of dentistry, and probably not what we shall require, which is a series of practical text-books. If there is a sentiment in favor of the movement to provide first-class textbooks, the next thing to do is to go to work to get them up; but it is a task that cannot be hurried. Such a system of books would make the teaching in the different colleges uniform, and would put money enough into the hands of the publishers to insure the prosecution of the work. It would be a man of considerable temerity who would undertake the preparation of a work on operative dentistry, and the probabilities are that when completed the profession would have taken a step a long way in advance of its teachings. But we must have something, and the best thing we can do will be to get up the best we can.

After discussion, on motion of Dr. Guilford, a committee of five, consisting of Drs. Abbott, Winder, Ingersoll, Guilford, and Fillebrown, was elected to take the subject into consideration and prepare suggestions as to the general scope and plan to be followed in the preparation of a series of dental text-books.

Dr. Abbott offered the following resolution, which was adopted and referred to the Executive Committee:

*Resolved*, That a standing committee on schools be elected, whose duty it shall be to ascertain as far as practicable the workings of all dental schools in this country and Europe, and be required to furnish information to the dean or secretary of any college when desired and to report in writing at each meeting of this association.

Dr. Truman offered the following, which was adopted:

*Resolved*, That the dean of each school be required to furnish the executive committee with the exact character of the intermediate examination, and whether any of them are final.

Dr. Truman offered a resolution that the winter terms of all dental colleges members of this association shall be at least seven months in duration. On motion of Dr. Abbott referred to the representatives of the different colleges with the request that they report upon it next year.

Dr. Guilford was appointed as a committee to codify the rules adopted by the association and prepare them for publication in the annual announcements of the colleges.

Dr. Fillebrown, secretary of the committee on text-books, read the report, stating that in the judgment of the committee text-books are needed on the following subjects: Oral Surgery; Dental Pathology and Therapeutics; Operative Dentistry and Orthodontia; Dental Chemistry and Metallurgy; Dental Prosthesis. Books on other subjects seem to be very well provided for at present. The report recommends that committes be appointed to solicit the writing of such books and to examine the manuscript and if found acceptable to authorize their publication, as text-books on these subjects, with the indorsement of this association, that the publication of the various books shall be under the supervision of committees composed of the professors in the colleges of this association of the particular branch of study to which the book is devoted, or such persons as the faculties may select; that the committees shall have power to solicit writers for the subjects named and to require the books to be written upon a plan acceptable to the committee and that the final copy be submitted to every member of the committee and unless it receives the approval of at least three-fourths of the whole committee it shall not be considered approved; that each writer shall be expected to retain the complete ownership of his manuscript and to publish at his own expense and risk.

The report was adopted and the chairmen of the committees on publication were appointed, as follows: "Oral Surgery," T. W. Brophy; "Dental Pathology and Therapeutics," James Truman; "Operative Dentistry and Orthodontia," Thos. Fillebrown; "Dental Chemistry and Metallurgy," A. O. Hunt; "Dental Prosthesis," S. H. Guilford.

The following officers were elected for the ensuing year: C. N. Peirce, president; R. B. Winder, vice-president; H. A. Smith, secretary; A. W. Harlan, treasurer, Frank Abbott, James Truman, J. Taft, executive committee; Frank Abbott, James Truman, R. B. Winder, committee to decide questions arising before the next meeting.

Adjourned.

---

### NATIONAL ASSOCIATION OF DENTAL EXAMINERS.

The National Association of Dental Examiners held its fifth session in the Park Theatre, Niagara Falls, commencing Monday, August 2, 1886, President J. Taft in the chair.

The following State boards were represented: Ohio, by J. Taft, H. A. Smith, and F. H. Rehwinkel; Illinois, by Geo. H. Cushing; Michigan, by A. T. Metcalf, F. W. Clawson, and G. S. Shattuck; California, by S. W. Dennis: Pennsylvania, by S. H. Guilford, W. E. Magill, and E. T. Darby; New Jersey, by Fred. A. Levy; Iowa, by J. T. Abbott; Maryland, by T. S. Waters; Louisiana, by Joseph Bauer; Indiana, by S. B. Brown; Wisconsin, by Edgar Palmer.

Officers were elected for the ensuing year as follows: J. Taft, president; H. A. Smith, vice-president; F. A. Levy, Orange, N. J., secretary and treasurer.

